# Targeted Lipidomics in *Drosophila melanogaster* Identifies Novel 2-Monoacylglycerols and *N*-acyl Amides

**DOI:** 10.1371/journal.pone.0067865

**Published:** 2013-07-11

**Authors:** Giuseppe Tortoriello, Brandon P. Rhodes, Sara M. Takacs, Jordyn M. Stuart, Arjun Basnet, Siham Raboune, Theodore S. Widlanski, Patrick Doherty, Tibor Harkany, Heather B. Bradshaw

**Affiliations:** 1 Psychological and Brain Sciences, Indiana University, Bloomington, Indiana, United States of America; 2 Division of Molecular Neurobiology, Department of Medical Biochemistry and Biophysics, Karolinska Institutet, Stockholm, Sweden; 3 Department of Chemistry, Indiana University, Bloomington, Indiana, United States of America; 4 Wolfson Centre for Ageing-Related Diseases, King’s College London, London, United Kingdom; 5 European Neuroscience Institute, University of Aberdeen, Aberdeen, United Kingdom; Governmental Technical Research Centre of Finland, Finland

## Abstract

Lipid metabolism is critical to coordinate organ development and physiology in response to tissue-autonomous signals and environmental cues. Changes to the availability and signaling of lipid mediators can limit competitiveness, adaptation to environmental stressors, and augment pathological processes. Two classes of lipids, the *N*-acyl amides and the 2-acyl glycerols, have emerged as important signaling molecules in a wide range of species with important signaling properties, though most of what is known about their cellular functions is from mammalian models. Therefore, expanding available knowledge on the repertoire of these lipids in invertebrates will provide additional avenues of research aimed at elucidating biosynthetic, metabolic, and signaling properties of these molecules. *Drosophila melanogaster* is a commonly used organism to study intercellular communication, including the functions of bioactive lipids. However, limited information is available on the molecular identity of lipids with putative biological activities in *Drosophila*. Here, we used a targeted lipidomics approach to identify putative signaling lipids in third instar *Drosophila* larvae, possessing particularly large lipid mass in their fat body. We identified 2-linoleoyl glycerol, 2-oleoyl glycerol, and 45 *N*-acyl amides in larval tissues, and validated our findings by the comparative analysis of Oregon-RS, Canton-S and w1118 strains. Data here suggest that *Drosophila* represent another model system to use for the study of 2-acyl glycerol and *N*-acyl amide signaling.

## Introduction

The fruit fly (*Drosophila melanogaster*) is one of the most important organisms in biomedical research with detailed knowledge available on its development and general physiology. The fat body of insects is the organ to control energy expenditure through the dynamic mobilization of energy reserves (glycogen and triglycerides) in adipocytes, analogous to mammalian white adipose tissue, to reflect the insect’s energy demands [1]. Insect adipocytes also maintain lipid reserves as cytoplasmic lipid droplets [2].

Lipids are structurally diverse molecules functionally underpinning energy storage, the structure of cell membranes, and intra- or intercellular signaling. The maintenance of lipid homeostasis in *Drosophila melanogaster* is critical to maintain growth [3], lifespan [4], movement control and reproduction, particularly during periods of restricted food availability or fasting [5]. In humans, perturbations of lipid metabolism and signaling are implicated in the pathogenesis of the most common and devastating illnesses, including diabetes, obesity, heart disease and neurodegeneration [6], [7], [8], [9].

Modernized lipid extraction and mass spectrometry techniques have provided the tools necessary for the field of lipidomics to exponentially expand during the past two decades [8], and led to the discovery of many small lipids with putative biological activity [10]. 2-Acyl glycerols and *N*-acyl amides have received growing interest given their widespread, tissue-specific and developmentally regulated roles in the mammalian body. Notably, the “endocannabinoids” 2-arachidonoyl glycerol (2-AG) and *N-*arachidonoyl ethanolamine (anandamide/AEA) are the most studied representatives of these two classes of molecules [11], [12], [13] given their control of energy homeostasis [9], [14], [15] at the periphery and synaptic neurotransmission in the nervous system [12], [16]. Although ubiquitously present in vertebrate species, neither molecule is produced by cholesterol auxotroph insects due to the lack of Δ-6/Δ-5 desaturases required to synthesize C20∶4/C22∶6 polyunsaturated fatty acids (PUFAs) [17], [18]. Accordingly, cladistic analysis combined with *in silico* functional mapping suggests that *Drosophila melanogaster* also lacks cannabinoid receptors required to transduce 2-AG and AEA signals [19], [20].

Nevertheless, *Drosophila melanogaster* express a limited number of co-evolved genes whose mammalian orthologs control endocannabinoid availability [19], [20]. Metabolic enzymes are rarely specific to generate a single product, giving rise to the possibility that *Drosophila* may contain and use hitherto unidentified signaling lipids. In particular, the fruit fly expresses an ancestral form of diacylglycerol lipase (“inactivation no afterpotential E”/InaE) [21], which may produce 2-AG congeners in the absence of arachidonic acid-containing diacylglycerol substrates. Therefore, we hypothesized that the repertoire of 2-acyl glycerols and 2-acyl amides produced by *Drosophila melanogaster* may be similar to those in vertebrates only lacking in the C20∶4/C22∶6 polyunsaturated fatty acid conjugates, and therefore, might emerge as a novel model to study the signaling properties of these classes of lipids. We used targeted lipidomics techniques previously described in mammalian tissues [22]–[27] to isolate 2-monoacylglycerols and *N*-acyl amide lipids from third instar larvae (LIII) of *Drosophila melanogaster*, when the fat body is particularly large in relation to the entire body mass [1]. We demonstrate the presence of 2-linoleoyl glycerol (2-LG), 2-oleoyl glycerol (2-OG), as well as 45 *N*-acyl amides, including 4 *N*-acyl ethanolamines that may represent the products or precursors of ancestral genes regulating endocannabinoid signaling in evolutionarily higher organisms.

## Materials and Methods

### Drosophila melanogaster Larvae

The wild-type *Drosophila melanogaster* strains Oregon-RS (#4269), Canton-S (#1), w1118 (#4605) and Cha-Gal4,UAS-GFP flies expressing green fluorescent protein in cholinergic neurons (#6793, all from the Bloomington Stock Center, Indiana, IN) were raised at 25°C with 12/12 h light/dark cycle. Flies were reared on standard medium (Nutri-fly Bloomington formulation, Genesee Scientific, San Diego, CA). LIII larvae (*n* ≥300/sample) were collected, rinsed twice in 50 mM phosphate-buffered saline, snap frozen in liquid N_2_, and stored at −80°C for lipid extraction. T1117, a fluorescent lipophilic dye (10 µM; Tocris, St. Louis, MO) whose target receptors [28] are not expressed in *Drosophila melanogaster* [20], was mixed in the media to localize dietary lipid accumulation.

### Analytical Standards and Reagents

AEA, 2-AG, 2-linoleoyl glycerol, *N*-palmitoyl ethanolamine (PEA), *N*-stearoyl ethanolamine (SEA), *N*-oleoyl ethanolamine (OEA), *N*-linoleoyl ethanolamine (LEA), *N-*docosahexaenoyl ethanolamine (DHEA), *N*-arachidonoyl glycine (NAGly), *N*-linoleoyl glycine (LinGly); *N*-oleoyl glycine (OlGly); and NAGly-d_8;_ and 2-AG-d_8_ were from Cayman Chemical (Ann Arbor, MI). 2-oleoyl glycerol was obtained from Avanti Polar Lipids (Alabaster, Alabama). All additional *N*-acyl amides were made in house as previously described [29]. HPLC-grade water and methanol were purchased from VWR International (Plainview, NY). HPLC-grade ammonium acetate was from Sigma-Aldrich (St. Louis, MO). C18 solid phase extraction and analytical (Zorbax eclipse XDB 2.1×50 mm reversed phase) columns were purchased from Varian (Harbor City, CA).

### Lipid Extraction

Lipids were extracted and partially purified as previously described in mammalian tissues [23], [25], [29], [30]. In brief, 40∶1 volumes of methanol were added to each sample followed by 2-AG-d_8_ and NAGly-d_8_ (10 µl of 100 pM). These deuterium-labeled compounds were used as internal standards to determine the extraction efficiency. Methanolic samples were covered with parafilm and left on ice and in darkness for ∼2 h. Samples on ice were then homogenized using a polytron for ∼1 min followed by centrifugation at 19,000 *g* at 24°C for 20 min. Supernatants were then collected and placed in polypropylene tubes. HPLC-grade water was added, making the final supernatant/water solution 25% organic. To isolate the compounds of interest partial purification of the 25% solution was performed on a Preppy apparatus assembled with 500 mg C18 solid-phase extraction columns. The columns were conditioned with 5 ml of HPLC-grade methanol immediately followed by 2.5 ml of HPLC-grade water. The supernatant/water solution was then loaded onto the C18 column, and then washed with 2.5 ml of HPLC grade water followed by 1.5 ml of 40% methanol. Elutions of 1.5 ml of 70%, 75%, 80%, 85%, 90%, 95%, and 100% methanol were collected in individual autosampler vials and stored at −20°C until analysis by mass-spectrometry.

### LC/MS/MS Analysis and Quantification

HPLC/MS/MS methods previously described were used for each of the 73 lipids analyzed here [24]–[26], [30]. Tables 1 and 2 provide the parent and fragment ions for each. With the exception of the 2-acyl glycerol and *N*-acyl ethanolamine species, which were analyzed using positive ion modes [H+], all other lipids were analyzed in negative ion mode [H−], likewise, as previously described [24], [30], [31]. Elutions were removed from −20°C storage and allowed to warm to room temperature while covered (∼20 min), vortexed for ∼1 min before being placed into the autosampler (Agilent 1100 series autosampler, Palo Alto, CA), and held at 24°C for LC/MS/MS analysis. 10–20 µl of the eluents were injected separately for each sample to be rapidly separated using a C18 Zorbax reversed-phase analytical column to scan for individual compounds. Gradient elution (200 µl/min) occurred under the pressure created by two Shimadzu 10AdVP pumps (Shimadzu, Columbia, MD). Next, electrospray ionization was accomplished using an Applied Biosystems/MDS Sciex API3000 triple quadrupole mass spectrometer (Applied Biosystems, Foster City, CA). Peak matching analysis in the Analyst software package that is associated with Applied Biosystems (Foster City, CA) mass spectrometers uses multiple reaction monitoring (MRM) mode in order to analyze the level of each compound present in the sample injection. In order for the analytical software to be able to accurate match peaks, synthetic standards were used to generate optimized MRM methods and standard curves for analysis. Additional analysis used was the product ion scan in which the parent ion was filtered in the first quadrupole and then all fragments were monitored to generate a complete fingerprint of molecular fragments.

**Table 1:** Mass spectrometry detection of 2-monoacylglycerols and 6 classes of *N*-acyl amides in Oregon-RS larvae.

Common Name	Parent ion [H+ or H−]	Fragment ion [H+ or H−]	Presence in Oregon-RS LIII
***2-*** **acyl glycerols**			
*2-*oleoyl glycerol	357.5	265.2	detected
*2-*linoleoyl glycerol	355.5	245	detected
*2-*arachidonoyl glycerol	379.3	287.5	n.d.
***N*** **-acyl alanine**			
*N*-palmitoyl alanine	326.5	88.09	detected
*N*-stearoyl alanine	354.55	88.09	detected
*N*-oleoyl alanine	352.53	88.09	detected
*N*-linoleoyl alanine*	350.52	88.09	detected
*N*-arachidonoyl alanine	374.5	88.09	n.d.
*N*-docosahexaenoyl alanine	398.56	88.09	n.d.
***N*** **-acyl ethanolamine**			
*N*-palmitoyl ethanolamine	300.29	62.1	detected
*N*-stearoyl ethanolamine	328.3	62.1	detected
*N*-oleoyl ethanolamine	326.3	62.1	detected
*N*-linoleoyl ethanolamine	324.3	62.1	detected
*N*-arachidonoyl ethanolamine	348.29	62.1	n.d.
*N*-docosahexaenoyl ethanolamine	372.6	62.1	n.d.
***N*** **-acyl GABA**			
*N*-palmitoyl GABA	340.54	102.1	n.d.
*N*-stearoyl GABA	368.58	102.1	n.d.
*N*-oleoyl GABA	366.57	102.1	detected
*N*-linoleoyl GABA*	364.54	102.1	detected
*N*-arachidonoyl GABA	383.57	102.1	n.d.
*N*-docosahexaenoyl GABA	412.59	102.1	n.d.
***N*** **-acyl glycine**			
*N*-palmitoyl glycine	312.26	74.2	detected
*N*-stearoyl glycine	340.3	74.2	detected
*N*-oleoyl glycine	338.3	74.2	detected
*N*-linoleoyl glycine	336.3	74.2	detected
*N*-arachidonoyl glycine	360.3	74.2	n.d.
*N*-docosahexaenoyl glycine	384.3	74.2	n.d.
***N*** **-acyl leucine**			
*N*-palmitoyl leucine	368.58	130.1	detected
*N*-stearoyl leucine*	396.63	130.1	detected
*N*-oleoyl leucine	394.61	130.1	detected
*N*-linoleoyl leucine*	392.6	130.1	detected
*N*-docosahexaenoyl leucine	440.64	130.1	n.d.
***N*** **-acyl methionine**			
*N*-palmitoyl methionine	386.62	148.2	detected
*N*-stearoyl methonine	414.64	148.2	n.d.
*N*-oleoyl methionine	412.65	148.2	detected
*N*-linoleoyl methionine*	410.64	148.2	detected
*N*-arachidonoyl methionine	434.66	148.2	n.d.
*N*-docosahexaenoyl methionine	458.68	148.2	n.d.

Parent and fragment ions for each of the lipids were reported. Note that arachidonoyl (C20∶4) and docosahexaenoyl (C22∶6) derivatives are not present in *Drosophila melanogaster*. Asterisks indicate lipids not yet identified in a biological system. *n.d*. indicates that the chromatographic/MRM match was not detected.

**Table 2:** Mass spectrometry detection of 6 classes of *N*-acyl amides in Oregon-RS larvae.

Common Name	Parent ion [H+ or H−]	Fragment ion [H+ or H−]	Presence in Oregon-RS LIII
***N*** **-acyl phenylalanine**			
*N*-palmitoyl phenylalanine	402.59	164.1	detected
*N*-stearoyl phenylalanine	430.65	164.1	detected
*N*-oleoyl phenylalanine	428.63	164.1	detected
*N*-linoleoyl phenylalanine*	426.61	164.1	detected
*N*-arachidonoyl phenylalanine	450.64	164.1	n.d.
*N*-docosahexaenoyl phenylalanine	474.66	164.1	n.d.
***N*** **-acyl proline**			
*N*-palmitoyl proline	352.53	114.12	detected
*N*-stearoyl proline	380.59	114.12	detected
*N-o*leoyl proline	378.31	114.12	detected
*N*-linoleoyl proline*	376.56	114.12	detected
*N*-arachidonoyl proline	400.58	114.12	n.d.
*N*-docosahexaenoyl proline	424.6	114.12	n.d.
***N*** **-acyl serine**			
*N*-palmitoyl serine	342.3	74	detected
*N*-stearoyl serine	370.3	74	detected
*N*-oleoyl serine	368.3	74	detected
*N*-linoleoyl serine*	366.27	74	detected
*N*-arachidonoyl serine	390.3	74	n.d.
*N*-docosahexaenoyl serine	414.3	74	n.d.
***N*** **-acyl tryptophan**			
*N*-palmitoyl tryptophan	441.63	203.1	detected
*N*-stearoyl tryptophan	469.68	203.1	detected
*N*-oleoyl tryptophan	467.67	203.1	detected
*N*-linoleoyl tryptophan*	465.65	203.1	detected
*N*-arachidonoyl tryptophan	489.67	203.1	n.d.
*N*-docosahexaenoyl tryptophan	513.69	203.1	n.d.
***N*** **-acyl tyrosine**			
*N*-palmitoyl tyrosine	418.59	180.18	detected
*N*-stearoyl tyrosine	446.65	180.18	detected
*N-*oleoyl tyrosine	444.63	180.18	detected
*N*-linoleoyl tyrosine*	442.61	180.18	detected
*N*-arachidonoyl tyrosine	466	180.18	n.d.
*N*-docosahexaenoyl tyrosine	490.66	180.18	n.d.
***N*** **-Acyl valine**			
*N*-palmitoyl valine	354.31	116.31	detected
*N*-stearoyl valine	382.6	116.14	detected
*N*-oleoyl valine	380.59	116.14	detected
*N*-nervonoyl valine	464.75	116.14	n.d.
*N*-linoleoyl valine*	378.58	116.14	detected
*N*-docosahexaenoyl valine	426.62	116.14	n.d.

Parent and fragment ions for each of the lipids were reported. Note that arachidonoyl (C20∶4) and docosahexaenoyl (C22∶6) derivatives were absent from *Drosophila melanogaster*. Asterisks indicate lipids not yet identified in a biological system. *n.d*. indicates that the chromatographic/MRM match was not detected.

### Data Analysis

All lipid analytes were identified using synthetic standard matching chromatographic peaks coupled to matching MRM scans as previously described [24], [25], [27], [30]. The standards provided a reference for the retention times and mass fingerprint by which the analytes were compared. They allowed the identification of the specific precursor ion and fragment ion for each analyte to enable their isolation. In addition, some more abundant lipids were further verified using product ion scans. Quantitation of analytes was calculated by using a combination of calibration curves of the synthetic standards obtained from the Analyst software and recovery adjustments using deuterium-labeled internal standards. These processes cumulatively were sufficient to accept the molecular identity of the lipid species with high confidence. We ensured the robustness of our measurements, including strain comparisons, by collecting *n* ≥300larvae/sample from 3–4 cohorts of independently reared larvae and analyzing them in triplicate. Quantitative data were expressed as the means +/− sem of triplicate measurements, and statistically evaluated using Student’s *t*-test. *P*<0.05 was considered statistically significant. Average recoveries of deuterium-labeled compounds across these samples were 91% with a standard deviation of 5%.

## Results

### Identification of 2-acyl Glycerols

**Figure 1:**
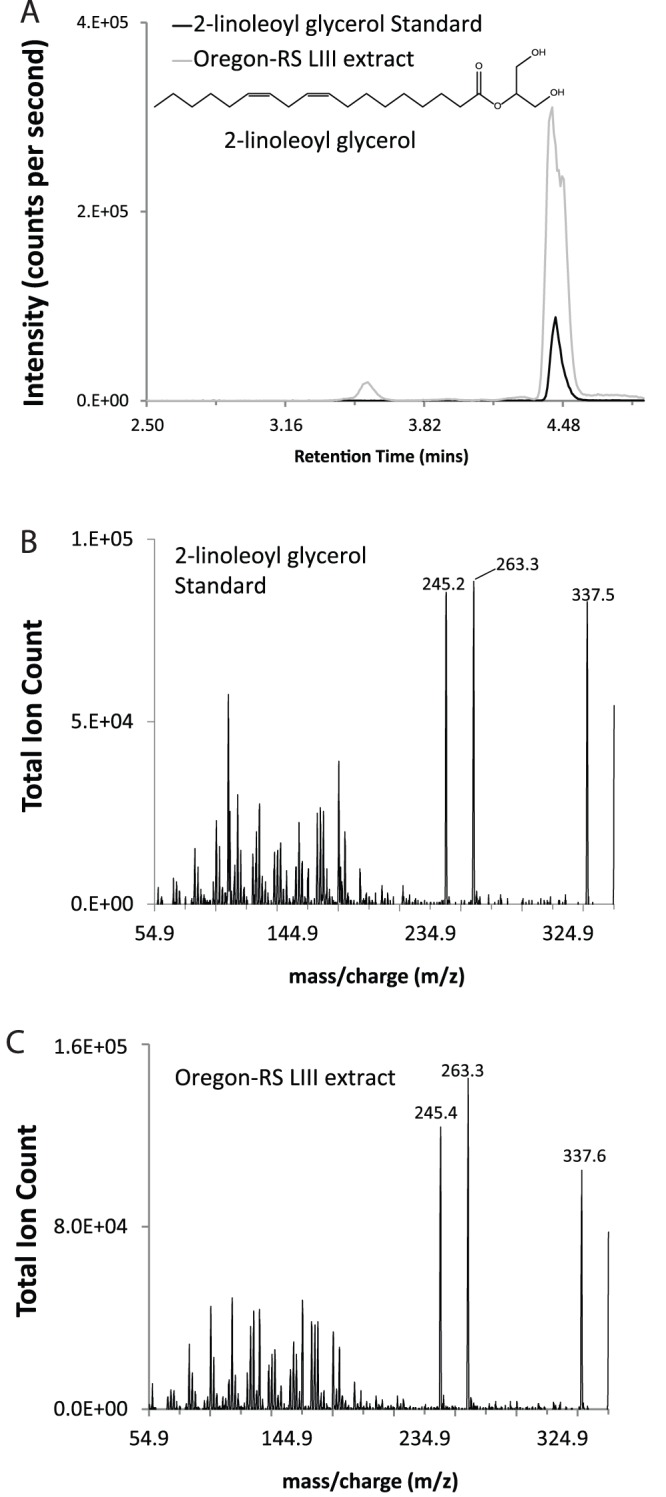
Identification of 2-linoleoyl glycerol (2-LG) from third instar Oregon-RS larvae. (A) Matching chromatographic peaks of synthetic 2-LG standard and LIII *Drosophila* lipid extracts. (B) Product ion scan for the parent ion 355.5 [H^+^] using the 2-LG standard. (C) Product ion scan for the parent ion 355.5 [H^+^] using the lipid extract from *Oregon-RS Drosophila* larvae.

2-linoleoyl glycerol (2-LG) and 2-oleoyl glycerol (2-OG) were detected in lipid extracts from the whole body of third instar (LIII) Oregon-RS larvae (Fig. 1, Table 1). As shown in Fig. 1A, the overlay of chromatograms of synthetic 2-LG and LIII Oregon-RS extracts indicated matching retention times. Similarly, product ion scans of synthetic 2-LG and LIII lipid extracts (Fig. 1B, C) illustrate identical molecular species, demonstrating a match between the synthetic standard and the compound isolated from *Drosophila melanogaster* larvae. Product ion scans of the standard (Fig. 1B) and the extract (Fig. 1C) show identical fragment ions as the most abundant ions in the scan. These spectrum scans (Fig.1B,C) were performed under the maximum peak height at the same retention time of the chromatographic peaks determined in the MRM methods. The presence and molecular identity of 2-OG were verified by the same protocol (Table 1). In contrast, matching chromatograms were not observed for the MRM or product ion scan methods of 2-AG in the extract confirming earlier findings that arachidonoyl metabolites are not natively present in *Drosophila*.

### Identification of *N*-acyl Amides

**Figure 2:**
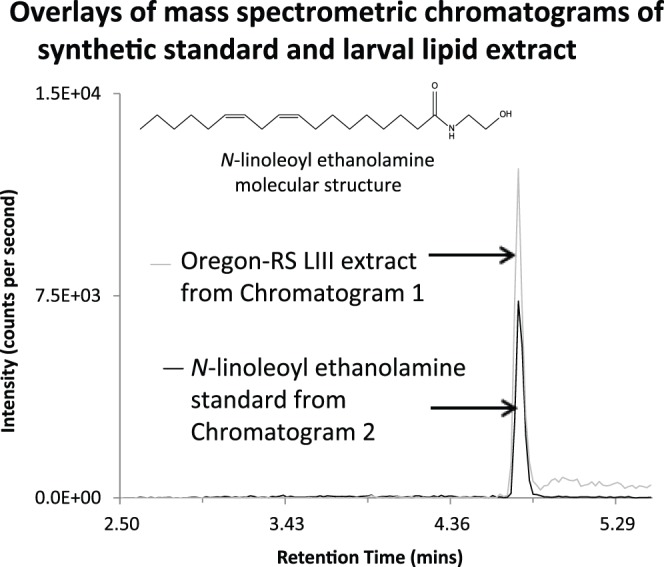
Overlay chromatograms from mass spectrometric data comparing synthetic standards and lipid extracts from third instar Oregon-RS larvae. This is an example of the type of data that are compared via the analytical software to determine chromatographic/mass spectrometric matches from unknown samples to the synthetic standards. Here, the example of the HPLC/MS/MS chromatogram generated using the analytical method for the synthetic standard of *N*-linoleoyl ethanolamine is overlaid with a partially purified lipid extract from third instar Oregon-RS larvae. Matching retention times and chromatographic peaks that are generated with the mass spectrometric match of the MS/MS of the parent/fragment ion pair associated with the synthetic standards provide clear evidence that the same compound exists in the extract sample.

**Figure 3:**
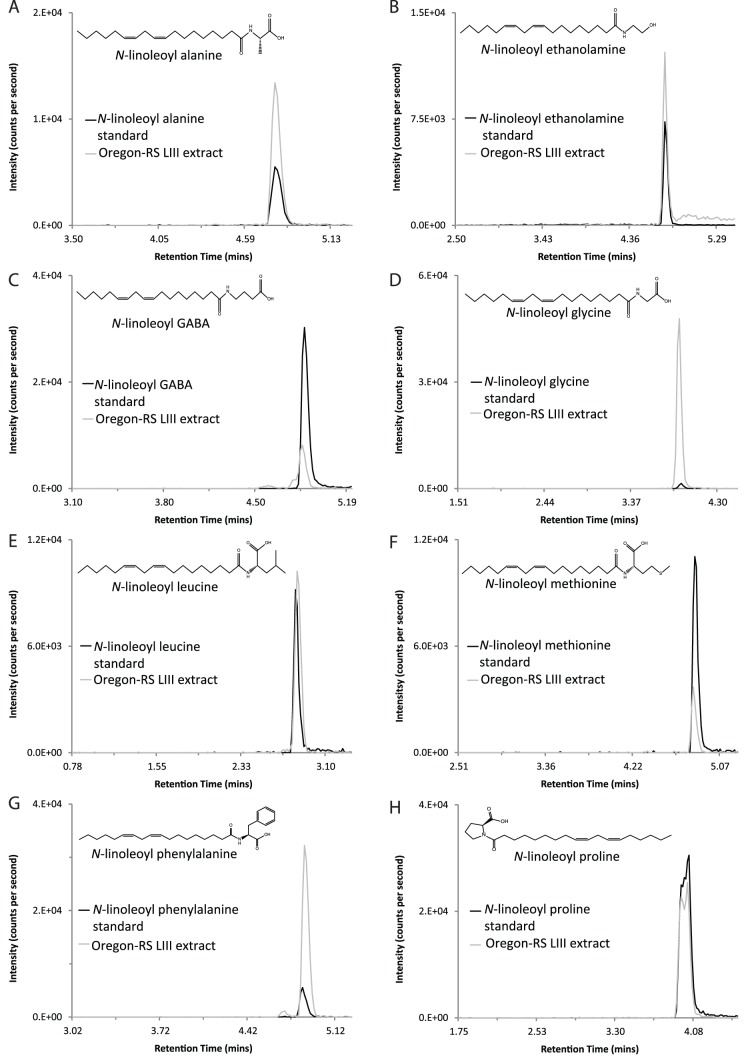
Representative overlay chromatograms of eight *N*-linoleoyl amides isolated from third instar Oregon-RS larvae. Lipid extracts analyzed using HPLC/MS/MS were from Oregon-RS *Drosophila* larvae raised on base diet formulation. A–H) Chromatographic overlays of two individual scans show identical retention times for the standards (in black) and analytes isolated from *Drosophila* lipid extracts (grey). Insets show the molecular structure of each lipid. (3B is a replica of the example given in Fig. 2. It is shown here to provide a standard for how it relates to the other analogous compounds.).

**Figure 4:**
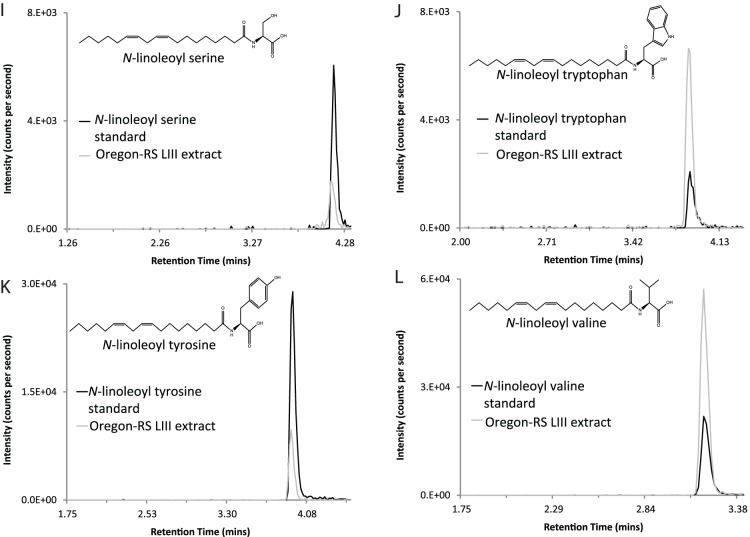
Representative overlay chromatograms of four *N*-linoleoyl amides isolated from third instar Oregon-RS larvae . Lipid extracts analyzed using HPLC/MS/MS were from Oregon-RS *Drosophila* larvae raised on base diet formulation. I–L) Chromatographic overlays of two individual scans show identical retention times for the standards (in black) and analytes isolated from *Drosophila* lipid extracts (grey). Insets show the molecular structure of each lipid.

Using analytic methods that had shown a majority of the *N*-acyl amides screened in mammalian species [23], [29], [30], our results identified 45 of the 70 *N*-acyl amide chromatographic matches in LIII Oregon-RS extracts. An example of these matches is shown in Figure 2 in which the MRM chromatogram of the *N*-linoleoyl ethanolamine standard is overlaid onto the MRM chromatogram of the LIII Oregon-RS extract using the same analytical parameters. These data illustrate pictorially the matching techniques that the Analyst software uses to determine “best fit” parameters for unknowns in relation to the standards. Figure 2 also shows an image of the molecular structure for *N*-linoleoyl ethanolamine. This is provided for each of the examples shown in Figures 3 and 4 as well to illustrate the diversity and yet, similarity of the *N*-linoleoyl amides identified here and *N*-acyl amides in general. This example, together with the together with the chromatography profiles depicted in Figures 3 and 4 are exact overlays and, therefore, the retention times shown in each demonstrate the degree of matching for the LIII larval extracts and the standards. Importantly, 8 of the *N*-linoleoyl amide conjugates had not been previously identified in biological tissues as evidenced by literature searches and the latest list of biologically identified lipids in the LIPID MAPS database located at the following URL: (http://www.lipidmaps.org/data/structure/LMSDSearch.php?Mode=ProcessClassSearch&LMID=LMFA08), and therefore, represent novel additions to the *N*-acyl amide family. Although PUFAs like C22∶6 (docosahexaenoyl) and C20∶4 (arachidonoyl) ethanolamines were not detected, we found C16∶0 (palmitoyl); C18∶0 (stearoyl); C18∶1 (oleoyl), and C18∶2 (linoleoyl) ethanolamines, which are all structural analogs to endogenous cannabinoids [32]. The same finding replicated with all other *N*-acyl amide conjugates screened in that all conjugates to each of the 4 acyl chains (C16∶0, C18∶0; C18∶1; C18∶2) with alanine, ethanolamine, glycine, leucine, phenylalanine, proline, serine, tryptophan, tyrosine, and valine were detected (Tables 1, 2). The only exceptions were that C16∶0 and C18∶0-GABA as well as C18∶0 methionine were not detected. However, the other conjugates for these amines were detected (Tables 1 and 2).

### Comparative Lipidomics in Three “Wild-type” Strains of *Drosophila melanogaster*

**Figure 5:**
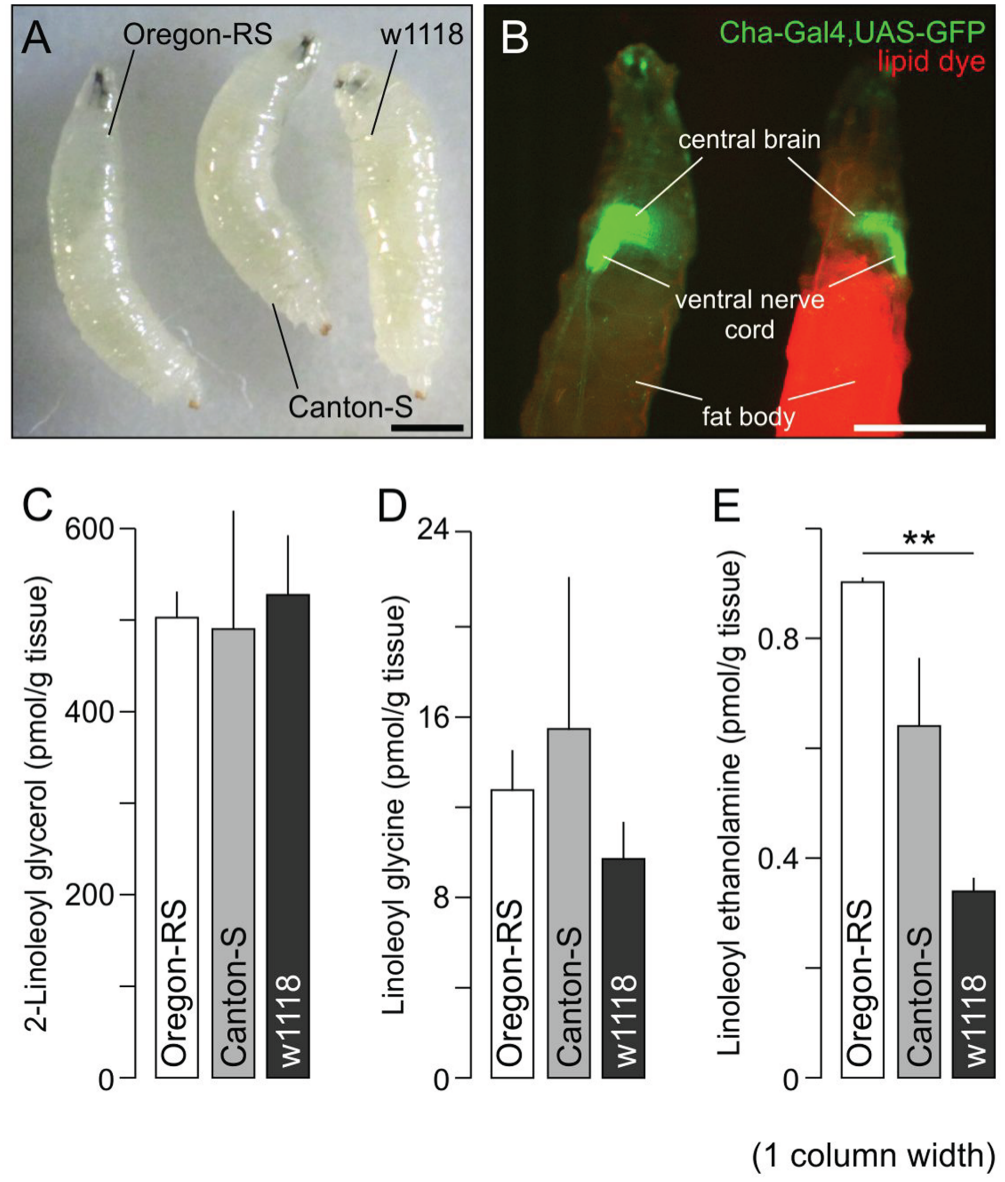
Comparative lipidomics in Oregon-RS, Canton-S and w1118 strains. (A) Third instar larvae (LIII) were reared simultaneously and photographed to demonstrate the lack of gross bodily differences. (B) T1117, a lipophilic dye without known receptors in *Drosophila melanogaster* [28], was used to localize the accumulation of dietary lipids. The Cha-Gal4 driver in combination with the UAS-GFP reporter was used to reveal both the central and peripheral cholinergic system in LIII. Note that the fat body is the primary site of lipophilic dye accumulation. (C–E) Comparative lipidomics in the three wild-type strains revealed comparable 2-LG and *N*-linoleoyl glycine, but not *N*-linoleoyl ethanolamine (*p* = 0.0017 Oregon-RS *vs*. w1118; (E) contents. Scale bars = 1 mm.

In addition to the Oregon-RS strain, Canton-S and w1118 flies are the most common laboratory stocks. These genetic backgrounds are widely used to engineer mutants and transgenic lines. During larval stages, Drosophila deposit excess energy from dietary food as lipids in the fat body. This is physiologically required since Drosophila cease food intake at the pupal stage, when they rely on stored lipids to fuel metamorphosis [33], [34]. Although neither the size nor the organ system anatomy of these strains is grossly different (Fig. 5A), with dietary lipids primarily accumulating in their fat body (Fig. 5B), their lipid metabolism may still vary. Therefore, we determined the concentrations of representative lipids in LIII Oregon-RS, Canton-S and w1118 reared and processed in parallel (Fig. 5C–E). We find LIII of these strains to contain equivalent amounts of 2-LG (Oregon-RS: 502±29.24; Canton-S: 488±128.94 and w1118∶526±64.95 pmol/g tissue; Fig. 5C) and *N*-linoleoyl glycine (Oregon-RS: 12.7±1.81; Canton-S: 15.5±6.77 and w1118∶9.7±1.68 pmol/g tissue; Fig. 5D). In contrast, N-linoleoyl ethanolamine levels were markedly different between Oregon-RS and w1118 larvae (Oregon-RS: 0.90±0.001 *vs*. w1118∶0.34±0.024 pmol/g tissue; *p* = 0.0017; Fig. 5E).

## Discussion

Even phylogenetically ancestral organisms with limited tissue complexity can synthesize a broad variety of small lipids whose abundance, organ system distribution and physiological roles may be differentially controlled across developmental stages or tissues at the cellular and subcellular levels. *Drosophila melanogaster* is gaining momentum as a favored model organism in lipidomic studies given its evolutionary conserved regulatory processes, such as *melted* [35] and *adipose* [36], as well as the breadth of genetic tools available to manipulate tissue-specific lipid metabolism in this species [37]. In the current post-genomic era, metabolomics aimed to identify and functionally analyze bioactive lipids and their hierarchical signaling cascades (from respective receptors to transcriptional mechanisms) that might function endogenously in the fruit fly can provide novel molecular insights in the regulation of signaling pathways relevant for disease-oriented studies in mammals.

Mass spectrometry is a powerful analytical tool to identify lipids within living organisms [38]. Its high mass resolution approaches allow this methodology to be extremely versatile during the high-throughput identification and quantification of cellular lipidomes [39]. Here, using lipidomics methods developed in mammalian tissues we obtained an initial profile of 2-monoacylglycerols and *N*-acyl amides in LIII of a common *Drosophila melanogaster* strain. Our analysis was prompted by the paramount importance of 2-AG and AEA members of these lipid classes in nervous system function [16] and the maintenance of energy balance in mammals [14], [15], including humans. We confirmed previous data showing the lack of 2-AG and AEA in *Drosophila melanogaster* [17]. We attribute this to be the direct consequence of the lack of arachidonic acid [18] in *Drosophila*, an essential fatty acid typically esterified in membrane phospholipids and a critical substrate for eicosanoid biosynthesis [40].

2-LG production in *Drosophila*, in which a linoleoyl group substitutes the arachidonoyl group of 2-AG, may provide metabolic alternatives in signaling networks otherwise triggered by 2-AG in vertebrates. Since 2-LG is unable to directly activate mammalian cannabinoid receptors but can potentiate the activity of other endocannabinoids, including 2-AG [41], our findings suggest that appropriate receptor families must have co-evolved in *Drosophila* to confer signal efficacy at the cellular level. Though focused on the metabolism of 2-AG, recent work by the Cravatt group demonstrated clear links in 2-AG and prostaglandin metabolism [42], [43]. Given the abundance of 2-LG in *Drosophila* tissue, it would be important to pursue any potential metabolic connections with oxygenated metabolites of both 2-LG and linoleic acid. Therefore, the fruit fly may be a helpful model to gain insights in the metabolism and signaling of 2-LG and 2-OG, which are present in the mammalian brain [44] and can inhibit fatty-acid amide hydrolase and monoacylglyecerol lipase [45], endocannabinoid degrading enzymes.

The lack of arachidonic and other long-chain fatty acids in *Drosophila melanogaster* led to the hypothesis that alternative PUFAs, such as linolenic acid, may control excitation at *Drosophila* photoreceptors [18], [46], although any direct evidence of this or another PUFA is still lacking, and indirect signaling pathways are being hypothesized [47]. Therefore, the discovery of more PUFA derived molecules in *Drosophila* may provide additional avenues of study for these mechanisms. Here, we also identified 2-OG, which is a ligand of GPR119 regulating incretin release from human intestines [48]. Cumulatively, our data warrant future functional studies aimed at dissecting *Drosophila*-specific receptors and cellular responses to 2-LG and 2-OG. These studies may be particularly exciting since they can also address the involvement of the *sn*-1-diacylglycerol lipase proto-ortholog (InaE, CG33174) in generating signal lipid diversity and whose activity is required to evoke physiological responses at TRP channels in *Drosophila* photoreceptors [21].

Multiple species of 12 subclasses of *N*-acyl amides were also identified in the Oregon-RS strain (Tables 1, 2). Eight of these *N*-acyl amides had not yet been identified in any biological tissue; however, unpublished data from our lab demonstrated that each are also present in mammalian tissue, suggesting an evolutionary overlap of the production of these lipids that likely spans many phyla. Our understanding of the cellular functions of this class of lipids is just beginning, although the signaling properties and (patho-)physiological function of some of these *N*-acyl amides are known (e.g., *N*-oleoyl ethanolamine regulates food intake and body weight in vertebrates *via* PPARα [49]). Therefore, in-depth functional characterization of novel *N*-acyl amides may significantly advance existing knowledge in functional lipidomics/metabolomics both in *Drosophila* and mammals. Consistent with this notion, fatty-acid amide hydrolase (CG8839), the major enzyme degrading *N*-acyl amides, is expressed along the entire life-span and localizes to lipid droplets in *Drosophila* [50].

Drosophila is a widely used organism in laboratory practice. However, lipid composition and metabolism in Drosophila melanogaster are different from vertebrates given the fruit fly’s deficiency in enzymes required to synthesize C20∶4/C22∶6 PUFAs [17], particularly arachidonic acid as a precursor [18]. Here, we show that there is significant diversity in lipid production even amongst common laboratory Drosophila strains. However, caution is to be exercised when interpreting data from this invertebrate model since most studies have used inbred strains maintained at high density in spatially uniform environments (vials, bottles). Inbreeding causes this normally highly heterozygous species to become homozygous and to fix unconditionally harmful mutations. As a consequence, the diversity amongst common laboratory Drosophila strains in lipid production can reflect different levels of genetic heterogeneity due to inbreeding rather than adaptive coevolution with physiological significance. Therefore, the data shown here illustrates that Drosophila lipidomics can offer new understanding in the molecular physiology of small signal lipids and their contribution to metabolic disorders.
